# Auditing the socio-environmental determinants of motivation towards physical activity or sedentariness in work-aged adults: a qualitative study

**DOI:** 10.1186/s12889-016-3098-6

**Published:** 2016-05-26

**Authors:** Richard Keegan, Geoff Middleton, Hannah Henderson, Mica Girling

**Affiliations:** Research Institute for Sport and Exercise Science, Faculty of Health, University of Canberra, Haydon Drive, Bruce, ACT 2601 Australia; School of Sport and Exercise Sciences, College of Social Sciences, University of Lincoln, Lincoln, UK

**Keywords:** Motivational climate, Determinants, Ecological, Behavioral etiology, Interviews, Health behavior

## Abstract

**Background:**

There is a lack of understanding of work aged adults’ (30–60 years old) perspectives on the motivation of physical activity versus sedentariness. This study aims to: (1) identify which socio-environmental factors motivate physical activity and/or sedentary behavior, in adults aged 30–60 years; and (2) explore how these motivators interact and combine.

**Method:**

Fifteen work-aged adults who, were able to engage in physical activity (Mean age = 43.9 years; SD 9.6, range 31–59), participated in semi-structured interviews. Inductive content analysis was used to generate an inventory of socio-environmental factors and their specific influences on motivation towards physical activity or sedentariness.

**Results:**

Key socio-environmental agents found to influence motivation included: Spouse/partner, parents, children, siblings, whole family, grandchildren, friends, work-mates, neighbors, strangers, team-mates and class-mates, instructors, health care professionals, employers, gyms and health companies, governments, media and social media, cultural norms, and the physical environment. Mechanisms fell into five broad themes of socio-environmental motivation for both physical activity and sedentariness: (1) competence and progress; (2) informational influences, (3) emotional influences, (4) pragmatics and logistics, and (5) relationships. Similar socio-environmental factors were frequently reported as able to motivate both activity and sedentariness. Likewise, individual categories of influence could also motivate both behaviors, depending on context.

**Conclusion:**

The findings of this paper ‘unpack’ theoretical concepts into specific and targeted behavioral recommendations. The data suggested no simple solutions for promoting physical activity or reducing sedentariness, but rather complex and interacting systems surrounding work-aged adults. Findings also suggest that health professionals should be encouraged to support adults’ health by examining the socio-environmental motivational influences, or ‘motivational atmosphere’.

## Background

Low levels of physical activity, alongside increasingly unhealthy diets, are a leading cause of obesity, diabetes, lifestyle-related illness and death [1–5]. The cost of physical inactivity to the United States economy has been estimated at US$75 billion per annum [6, 7]. In Britain, the cost to the National Health Service has been estimated at £8.2 billion per annum [8], while the wider costs to the economy are estimated to reach £49.9 billion per year [9, 10]. Only 5 % of American adults reach the recommended levels of physical activity [11]. A recent systematic review reported that in studies surveying adults around the world, the proportion of women meeting the recommended amounts of physical activity varied from 2 % in Taiwan and Saudi Arabia to 81 % in Denmark. In men, the proportion ranged from 4 % in Brazil to 77 % in Sweden [12]. Within that systematic review [12], the typical ‘guideline’ levels of physical activity were reported to be either ‘exercise six times in the previous 2 weeks’ or 30 min per day of moderate physical activity 5 days per week [12, 13]. Physical inactivity has been shown to be responsible for 5.3 million deaths per year worldwide, and if inactivity decreased by only 10 %, half a million deaths could be averted every year [14].

Research to promote physical activity often focuses on children and young adults (for example, adults are often classified as 17–30 years of age [12]) and older adults (often over 60 years old [15]). The vast majority of children attend schools, which provide a consistent (often mandatory) opportunity to engage in physical activity, in the form of physical education lessons [16, 17]. By contrast, older adults tend to more frequently attend primary healthcare settings for diagnosis and treatment, which then constitutes a strong opportunity to evaluate and promote physical activity. In many cases, physical activity represents a good management strategy for managing and preventing health problems experienced by older adults [18–21]. Working age adults, defined here as being between 30 and 60 years of age, are much less predictable in their movements, occupations, living arrangements and lifestyle choices [22, 23]. As a result, the population of work aged adults receives significantly less research attention. Nonetheless, research is needed to enable the generation of evidence-based guidelines to promote physical activity in this group. Such guidelines would help to both prevent inactivity related morbidity and mortality, as well as increasing quality of life, for a very large portion of a country’s economically productive population.

One core issue in overcoming this health burden is motivating busy work aged adults (30–60 years of age) to choose physical activity, when their work, homes and family life often readily facilitate sedentariness [24, 25]. Motivation has been defined as: “the hypothetical construct used to describe the internal and/or external forces that produce the initiation, direction, intensity and persistence of behavior” [26]. As such, motivation researchers frequently focus on the regulation of motivated behavior, as opposed to the observable outcomes such as effort, persistence, or task choice [27]. The motivational influence exerted by key social agents is referred to as the motivational climate [28], or motivational atmosphere [29]. Recent research has emphasized the benefits of concurrently examining influences from multiple socio-environmental agents, leading to a richer understanding of how these factors interact and combine to influence motivation [30, 31]. Additionally, these papers have illustrated the importance of resisting using a familiar and simplistic theoretical perspective *a priori* when viewing complex social phenomena [31–34]. By adopting such a theoretically agnostic approach [35] it is then possible to reflect back on compatibility with existing theories *a posteriori*, rather than allowing theory to determine what is examined, how, and how data is interpreted [35, 36]. This approach involves declining to adopt a single guiding explanatory framework *a priori;* instead engaging with the data in the full knowledge of existing theories (i.e. not naïve) but critically and effortfully seeking to avoid allowing one theory to steer data collection or interpretation: an open mind but not an empty head [37]. As such, inductive research is appropriately informed by existing theory but can also inform the development of new understanding [31, 38, 39]. This argument can be expressed as follows: theoretical frameworks can become simple classification systems for interpreting a complex phenomenon, undermining the emergence of new understanding. Rather, we may more fruitfully study the phenomena by capturing meaning that emerges from the data. Finally, since the social environment can simultaneously motivate physical activity and sedentariness, research is needed to assess these simultaneous influences [40, 41]. Recent research has recognized that individual participants are regularly exposed to concurrent motivational influences, and that decisions regarding health behaviors are made in these ‘relativistic’ terms, as opposed to ‘absolute’ terms (i.e., choosing one behavior over another, not in isolation [42–44]). Recognition of this approach necessitates seeking a range of participants and experiences, as opposed to groups that might be representative of either high or low physical activity (i.e., maximum variability sampling - [45]).

In light of the preceding considerations, the current study was designed to audit the sources and types of socio-environmental influences on motivation towards both physical activity and sedentariness in working age adults. Further, the study set out to organize these influences into a coherent framework. As such, the research questions for this study were:

1. Which socio-environmental influences, and social interactions, motivate physical activity and/or sedentary behavior, in work-aged adults (30–60 years old)?
2. How can these socio-environmental influences be organized and understood, in order to enable future research and practical intervention?

## Method

### Sampling strategy

Stratified sampling sought participants to create even/representative samples from activity levels (low, moderate, high), age, and occupation (unemployed, low skilled, high skilled/professional). Emails and intranet invitations were distributed through one UK university, one city council, and the exercise referral scheme operated by one primary healthcare trust (letter included in the ‘joining instructions’ for the scheme). Inclusion criteria were as follows: age between 30 and 60 years of age; and being physically able to engage in moderate to vigorous physical activity (MVPA). Of the 20 people who responded to the invitation to participate, two were excluded as they were unable to participate in physical activity. A further three were unable to arrange a convenient time for interview. Reflecting the geographical locale in the North-East of England, 14 of the participants were white European and one was Hispanic. As demonstrated in Table 1, the stratified sampling ensured a range of activity levels, ages and occupations, and the screening interview also ensured that participants were ‘information rich’ [46–48]. The sampling of a relatively wide range of activity levels and backgrounds – relative to the geographical area – was deliberate, and informed by the ‘maximum variability sampling’ strategy for qualitative research [45]. Achieving a range of ages, backgrounds and physical activity levels was one consideration that informed the decision to end recruitment (along with data ‘saturation’ – see below) [46–48].

**Table 1:** Summary of the participants recruited into the study

Participant number	Age (yrs)	Gender (F/M)	Occupation	Diagnosed health issues	Unique-Identifier	Estimated minutes of MVPA per week in last month (>5 METs)	Self-reported PA participation
1	45	F	Registered Nurse (senior)		F-45-Nurse	120	Gym, 3x/week - Treadmill walking, stationary cycling
2	35	F	Unemployed FT carer to disabled son		F-35-Carer	180	Gym, 3x/week – 30 min cardio plus resistance weight training
3	59	F	Unemployed	Myalgic Encephalomyelitis (ME)	F-59-Retired	50	Gym 2x/week – Yoga, Treadmill, cycle, swim
4	57	F	Retired. Volunteers in a hospice		F-57-Retired	90	Swimming once per week (1 h), looking after grandson once per week
5	43	F	Teaching Assistant and Foster Carer		F-43-Teaching-Assistant	120	Walking to work, participating in 2 PE lessons per week
6	47	F	Novel writer		F-47-Novellist	120	Walking to shops, housework, gardening
7	31	M	Sport Centre Manager.	Sprained Ankle	M-31-Sport-Centre-Manager	0	Badminton, Basketball (120/210 pre injury)
8	31	M	Office worker		M-31-Office-worker	240	2 games of rugby per week, 1 h of training
9	34	M	Technical Officer		M-34-Office-worker	0	Long walks at weekends
10	49	F	Sales Assistant		F-49-Sales-Assistant	0	Walking to and from work, approx. 2 h/week total
11	49	M	Office Manager		M-49-Manager	240	Cycling, 3–6 times per week, 20–25 miles each time
12	57	F	Office Worker		F-56-Office-worker	0	Dog Walking
13	49	M	Office Manager		M-49-Manager	150	Jogging twice per week, 8–10 miles, 75 mins each
14	36	M	Warehouse Supervisor		M-36-Warehouse-supervisor	180	Jogging twice per week, 12 miles 90 mins per run
15	37	M	Warehouse Supervisor		M-37-Warehouse-supervisor	0	Walking 3–4 times per week, approx. 60 mins each

### Design

A qualitative exploration of factors that motivate either physical activity or sedentary behaviors was undertaken using in-depth one-to-one interviews with work aged adults. Interviews were deployed to encourage participants to explore issues in-depth, and in their own language [49, 50]. A critical realist philosophical stance was adopted [29, 33, 38, 39], based on the assumption that the underlying intransitive ‘reality’ being studied is complex, but contains some consistencies that may be perceived by different observers (i.e., the participants). In light of this attempt to examine common or recurring socio-environmental influences, methodologies that would prioritize the individual’s unique lived experience (such as phenomenology and ethnography) were not the focus of this study [51–54]. That is not to say, however, that such approaches would be irrelevant or unhelpful for different research questions or philosophical assumptions. Consequently, participants were asked to describe lived experiences of real events, as they recalled them, and to expand on their explanations of how particular behaviors or attributes affected their motivation. In line with the assumption that all participant’s lives contained: (i) multiple concurrent experiences; that could motivate (ii) both physical activity and/or sedentariness; all participant were asked to discuss both outcomes [43, 44]. Interviews took place at the university campus, at participants’ offices, or over the telephone for those where face-to-face meetings were impractical. A semi-structured interview guide was deployed, based on those used in recent similar studies [29, 38]. After a brief introduction, the main questions assessed the influences of socio-environmental influences on motivated behaviors: defined as effort, persistence, task choice, focus, and enjoyment [55].

### Ethics

The study obtained ethical clearance from the University of Lincoln, College of Social Sciences ethics committee. Prior to beginning the interview, each participant gave full informed consent – in writing - for their data to be collected (audio recorded), stored (electronically), and published in any subsequent reports. There were no competing interests.

### Procedure

**Fig. 1:**
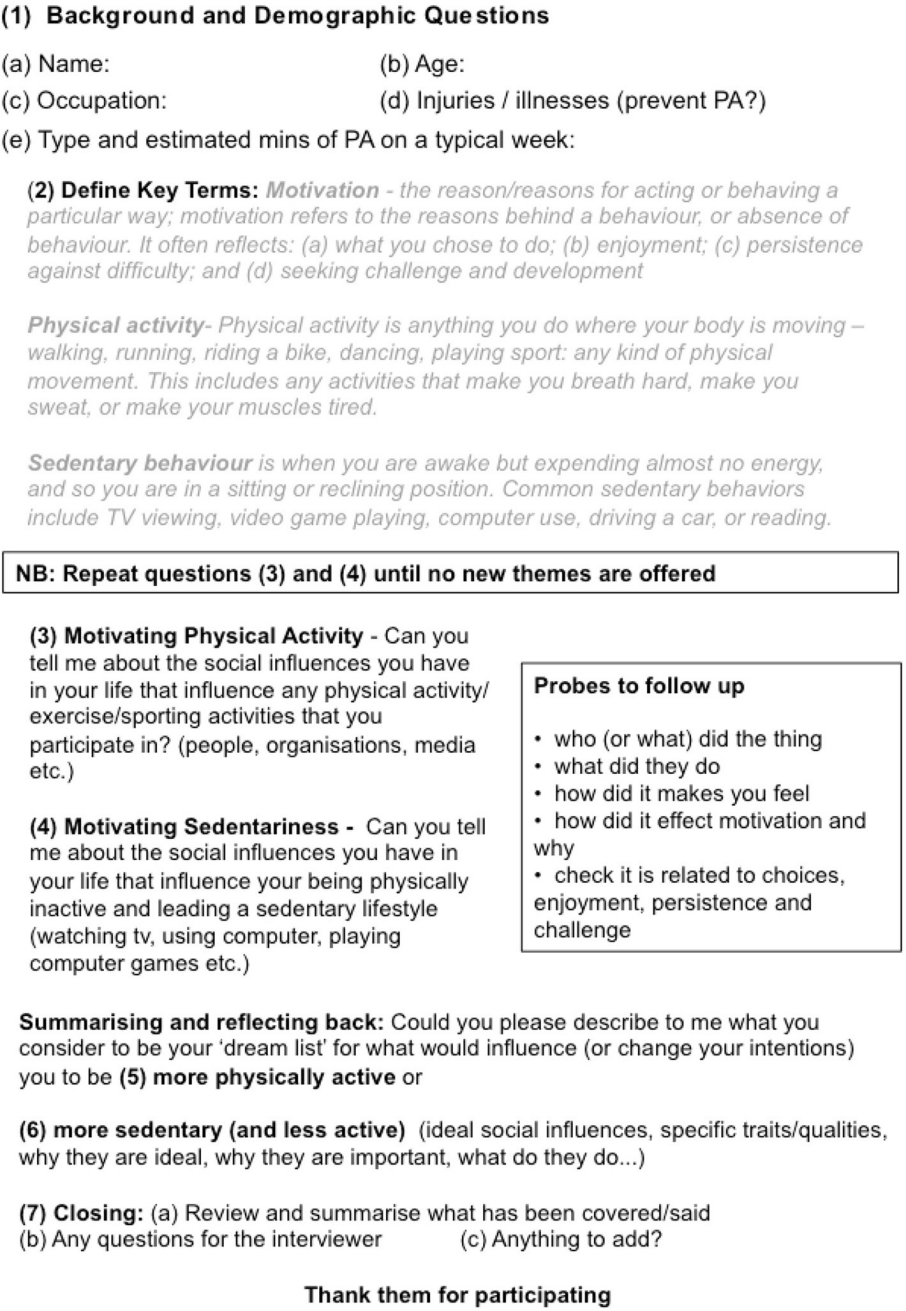
Interview guide that was used in this study. Note that questions 3–6 could be asked in a flexible order at the discretion of the interviewer

A single interviewer who had been specifically trained for this study contacted respondents to perform the screening interview and, if suitable, the full interview. Interviews were conducted face-to-face (*n* = 11), and by phone (*n* = 4), at which point the research team were in agreement that saturation was occurring in the data (i.e., key themes were being repeated and very few new concepts were being reported [56]). All interviews were conducted by the same interviewer, trained in qualitative methods (fourth author) and supported by an experienced qualitative researcher (first author). The interviewer was required to ask the same questions to each participant, but not always in the same order, to reduce the influences of question order or fatigue (see Interview Guide; Fig. 1).

At the beginning of each interview, the interviewer defined motivation, physical activity and sedentary behavior (see Fig. 1 - Interview Guide). The definition of motivation provided in the introduction was also explained. The interview questions centered around: (i) current levels of physical activity; (ii) who/what are the socio-environmental influences on motivation towards your physical activity, or sedentariness?; (iii) in relation to each socio-environmental influence, what have they done to facilitate/enhance your motivation towards physical activity, or sedentariness?; and (iv) what would need to change to significantly increase (or decrease) your motivation towards physical activity? The interview proceeded differently every time in response to the topics raised by participants. Participants were allowed to respond freely and, if questions intended for later in the interview were discussed, this was permitted. Impromptu probes were generated to explore themes and new questions that arose during interviews. Thus, while the interview was structured, there was flexibility within it to allow greater depth of exploration.

The interviewer audio transcribed interviews verbatim as soon as possible after they occurred, and the lead analyst began the analysis process immediately, in order to feed any new insights into subsequent interviews. This immediate transcription and analysis also informed the decision regarding when saturation was reached. Two qualified health researchers (second and third authors) assisted in the recruitment process by connecting the research team to important networks (local council, local exercise referral scheme). They also played a key role as critical friends in the consensus validation and peer debriefing aspects of the analysis [57].

### Data analysis

An eight-step analytic procedure [39] was implemented to prepare and analyze the data: (a) transcribe interviews verbatim (yielding 134 pages of single-spaced text, 58,200 words); (b) read and re-read transcripts for familiarization; (c) tag each quote as either concerning physical activity versus sedentary behavior – for the purpose of this analysis quotes that discouraged physical activity were classified under motivating sedentariness; (d) perform a thorough inductive content analysis, moving recursively between creating tags (“open coding”), creating categories (“focused coding”), and organizing categories into higher themes, using constant comparison and critical reflection to guide analysis [58]. This analysis was approached using QSR NVivo qualitative analysis software [59]; (e) inter-rater checking of the coding was completed using a sample of transcripts. The research team compared the independent codings of three randomly selected manuscripts, concluding that codings were semantically consistent in 88 % of the cases, which is deemed acceptable [60]; (f) member checking - which consisted of returning manuscripts and analysis outcomes to original participants for checking (six responses). This process did not suggest any modifications to the study findings or analysis, although participants did express strong agreement with the study findings and expressed interest in seeing future studies; (g) an iterative consensus validation process was conducted with two members of the research team to ensure the integration of codings into particular categories made the most analytic sense (which particularly focused on the labeling of themes and the suitability of quotes/codes for being coded into those themes); and (h) a peer debrief was conducted with an expert researcher throughout the analysis as well as in review of the final analysis. This structured use of multiple sources of data, investigators and theoretical viewpoints is proposed to facilitate a triangulation of the subject matter, which is less susceptible to individual bias [61]. Within the analysis process, all identified codes represented the interpreted meanings of the participants’ responses, focusing on specific behaviors and attributes of socio-environmental influences. Some codes were directly named after the participants’ own words, whilst others were named after concepts existing in the literature that were representative. In the latter case, processes of private reflection, consensus validation and peer review were utilized to ensure that these codes and categories were represented in the data and no “forcing” occurred during the coding [34]. Using constant comparison processes [45], the recursive coding of properties, interactions and contexts/situations was carried out until no new information about a category seemed to emerge [56]. Space considerations prevent the full presentation of quotes and illustrations, but in an attempt to demonstrate the transparency and authenticity of the research, and numerous quotes offer the reader a sense of personally experiencing the phenomenon being studied and presented [62].

## Results

The analysis identified 308 raw themes coded into 38 categories and five higher dimensions. Two-hundred and one raw themes (37 categories) pertained to motivating physical activity, while 107 raw themes (23 categories) pertained to motivating sedentariness. Mirrored categories were also identified that motivated both physical activity and/or sedentariness in similar ways. These mirrored themes are highlighted in the below analysis. Five higher-order themes of socio-environmental motivators emerged: (a) competence and progress; (b) informational influences; (c) emotional influences; (d) pragmatics and logistics; and (e) relatedness and belonging. The following presentation of results is organized to reflect these five higher-order themes. Where quotations are provided, the participant’s reference and source of influence are indicated as follows _[Gender-Age-Occupation-SOURCE]_.

### Competence and progress

Within this higher-order theme, there were 12 categories identified, containing 41 raw themes. There were three instances of categories mirroring across both physical activity and sedentariness. Overall, this higher-order theme reflects the way that competence within tasks is evaluated and recognized.

#### Mirrored categories

Categories pertaining to social judgments motivated both physical activity and sedentariness. Fear of negative social judgments could motivate physical activity (e.g., fearing the nurse’s judgment):

When it was just me and I was trying to lose weight on my own I was like ‘Yeah whatever’. However, seeing her I’m like ‘You know what I’d better lose something because she’s a right battle-axe!”… She’s just, her general demeanor she’s quiet mean…. just going to somebody whose expecting me to lose that weight and not doing it: I don’t like the thought of that. _[M-31-Sport-Centre-Manager-PRACTICE-NURSE]_

Alternatively, this fear could also demotivate physical activity (e.g., fearing the judgment of new team-mates):

I think there’s also a fear factor as well. Say if you joined a five a side football team there’s the unknown and the fact that they would all be fit and very physically active and your all unfit and out-of-breath… the fear factor of looking a fool in front of your peers… which I know is a catch 22 because the longer you don’t do it the worse its going to get, but it probably is a de-motivating factor. _[M-37-Warehouse-Supervisor-TEAMMATES]_

Likewise, social comparisons could either motivate physical activity or sedentariness, depending whether once compared well or badly. For example: “We went along [to badminton] thinking it would be ok to have a knock around, and it was just full of these ultra-fit ultra-talented people who were really going for it… It was far too competitive there really”_[M-37-Warehouse-Supervisor-TEAMMATES]_. Finally, the suitability of the activities could either motivate PA or sedentariness, such that lack of enjoyable activities could lead to sedentariness, or having desirable opportunities available could motivate physical activity.

#### Non-mirrored categories

Six categories that motivated physical activity were not mirrored under promoting sedentariness. These included: (i) healthy competition (e.g., versus team-mates/opponents, siblings, the instructor etc.). An example would include: “She used to get me to race her and I mean I never won but it’s good… she needed to get her times down… and I did get better and better at it, and I do really miss it”_[M-34-Office-Worker-PERSONAL TRAINER]_; (ii) noticing and recording progress (e.g., phone apps, workmates, family etc.); for example: “I think the little thing I have on my phone, that tells me ‘You have burned this amount of calories’. It’s like, you know ‘Yeah I did this amount yesterday, I can be at least the same today’”._[F-47-Novellist-PHONE-APP]_; (iii) activities individualized to me (e.g., gym instructor, personal trainer); for example: “I’m actually still on week one because I’ve not been very well, but the trainer says you can stay on week one as long as you like. I’m planning to move to week two next week”_[F-45-Nurse-GYM INSTRUCTOR]_; (iv) public accountability (e.g., phone apps, social media); (v) realistic pace of progress (e.g., gym instructor, personal trainer, phone app); and (vi) showing me how to do it properly (e.g., gym instructor, personal trainer; sister).

### Informational influences

Within this higher-order theme, there were 15 categories were identified, containing 82 raw themes. There were five instances of categories mirroring across both physical activity and sedentariness. Overall, this higher-order theme pertained to the nature, content, consistency and packaging of information that motivates either physical activity or sedentariness.

#### Mirrored categories

Categories pertaining to consistency of messages could motivate either physical activity (if consistent) or sedentariness (if contradictory). Likewise, role models could either motivate physical activity or sedentariness (e.g., “He lost three stone in a year, and I was still fat and he was thin, and I got a bit jealous. And it suddenly clicked and I decided I wasn’t a lost cause”_[F-56-Offier-worker-PARTNER]_, *versus* “I’ve got a teenage son who’s 14, and his idea of being motivated is sitting on the laptop all day. It doesn’t matter what you say to him. Going to shops, getting out of the house… it’s like pulling your hair out”_[F-35-Carer-SON])_. Social norms could be created that would either motivate physical activity (e.g., “I think because of experiences and how I was brought up as a child… if I notice I’m getting out of breath going up stairs or playing in the park… I would make a real conscious effort [to get fit again]”_[M-34-Office-Worker-PARENTS]_, or sedentariness (e.g., “I’s just nice and warm, isn’t it? …TV, computer… cooking and those sorts of things… it’s just what we do as a family” _[F-49-Sales-assistant-FAMILY]_). Likewise, shocking images in the media (or government campaigns) could motivate those who wish to avoid ill health, but were also described as desensitizing people, or causing feelings of hopelessness. For example: “To be honest with you I do take them with a pinch of salt…. The message seems to change week-by-week, so I think there’s a kind of filtering [out] mechanism in my mind’ _[M-37-Warehouse-manager-MEDIA]._ Finally, health professionals could either motivate physical activity by offering specific and personally relevant ‘warnings and alarms’ regarding health, or reinforce sedentariness by not mentioning health at a consultation. For example: “Well my father’s got high blood pressure, but the doctor highlighted it to me… …The initial doctor I saw suggested I needed to lose weight… I lost half a stone… then I went on and lost another couple of stone”_[M-49-Manager-GENERAL PRACTITIONER],_*versus* “No, the doctor never said anything. Last time I went my blood pressure was fine”_[F-43-Teaching-Assistant-GENERAL PRECTITIONER]_.

#### Non-mirrored categories

Four categories that motivated physical activity were not mirrored under promoting sedentariness. These included: (i) ‘my job gives me an awareness’ (e.g., those who worked in health promotion etc.); (ii) personal assessment opened my eyes (e.g., exercise referral worker or personal trainers) – for example: “The first week I saw her she just said write down everything you eat and do, don’t change anything, and I did. And I was quite shocked really… Er, it sort of come from there and then”_[F-57-Retired-EXERCISE-REFERRAL-WORKER]_; (iii) raising awareness and making me think (e.g., media campaigns, posters in health centers) – which can be illustrated by the following quote: “Well there are a lot of programs on [TV] about obesity… I went to the doctor and she told me I was borderline obese… so yes it wasn’t quite ideal”_[M-49-Manager-MEDIA + GENERAL PRACTITIONER];_ and (iv) referrals and recommendations (e.g., between healthcare providers, or by friends to attend a specific activity) – for example: “I was talking to the nurse who was doing the checking my blood pressure, and before she said anything I said ‘Look ok is there anything [I can do]… any programs?’ and she said yes. So she referred me.”_[F-47-Novellist-PRACTICE-NURSE]_. One category was listed as motivating sedentariness only, which was labeled ‘too many temptations’. In this category, participants discussed the wide range of easily accessible opportunities to be sedentary, and the attention-grabbing, immediately rewarding nature of these sedentary activities. The following quote illustrates the theme:

Just too many temptations. It’s too easy just to stay in and game. It’s too easy just to go home, put the heating on and get comfy, have a couple of drinks… alcoholic drinks. You get used to it and you get in that kind of mindset. _[M-34-Office-Worker-PHYSICAL ENVIRONMENT + NORMS]_

### Emotional influences

Within this higher-order theme, there were nine categories identified, containing 55 raw themes. There were four instances of categories being mirrored across both physical activity and sedentariness. Overall, this higher-order theme pertains to the ways that the provision of emotional and moral support can motivate either physical activity or sedentariness.

#### Mirrored categories

Categories pertaining to doing a specific activity together could motivate either physical activity or sedentariness. For example, “Cycling with my husband, I’m not just cycling, I’m getting to chat to him: ‘This is what I’ve done today…’ For me, if it was just running for running’s sake then it wouldn’t happen”_[F-45-Nurse-HUSBAND]_, versus “I get up in the morning and watch [TV] with a cup of coffee, and he’ll spend some time on the computer and I spend a lot of time with him watching TV”_[F-57-Retired-HUSBAND]_. Likewise, constant prompting and reminding could motivate physical activity or, if viewed as too much, sedentary behaviors (e.g., “My partner bless her, soul, she might say ‘Oh let’s go and do something’ and I’m at the crossroads like ‘walk, do something…’ you know? I think it pushes me to [do it]”_[F-47-Novellist-PARTNER]_, versus “It would completely demotivate me. By going on and on…. The amount of times we’ve had arguments and I’ve had to sleep downstairs…”_[M-31-Sport-Centre-Manager-WIFE]_). Notably this ‘prompting’ could come from husbands/wives/partners, as well as children and parents, and even teammates missing a key player. Moral support and encouragement could motivate physical activity or its absence could steer towards sedentariness. For example where spouses, children or friends took an interest and showed approval, physical activity could be encouraged. In contrast, where these social agents showed disinterest or disapproval towards physically active pursuits, sedentariness was encouraged. Finally, altruistic behaviors such as looking after friends or family could both motivate physical activity (for example, taking grand-children to the park) or motivate sedentariness (for example, spending time with a depressed or immobile friend). An example of caring for another leading to sedentariness is illustrated in the following quote:

I knew him [friend] and he seemed quite jolly but underneath it he’s absolutely not… He was becoming a bit like a lost cause… He was spending a great deal of time at my house, but he’s extremely lethargic, he is very obese… and he loves watching TV, so you know, we would. It was just too much inactivity. Lots of nights watching loads of TV._[F-59-Retired-FRIEND]_

Hence, high quality relationships could both motivate physical activity and sedentariness depending on the context.

#### Non-mirrored categories

One category - allowing ‘me time’ - was listed as promoting physical activity but not sedentariness. Where spouses and family were willing to ‘release’ a parent to be active, or where teammates understood that a colleague wanted to be quiet and alone, this category was used to capture the behavior. This can be illustrated as follows:

In terms of pressures of running a home and sorting the children out… sometimes there’s not much time in the day to do exercise… If I can go running in the morning that means I can do it at times when there’s not a lot else going on, and the kids aren’t usually awake. By the time I get back and I’m done I’m ready to take on my responsibilities as a parent, and husband….”_[M-49-Manager-WIFE]_

### Pragmatics and logistics

This higher order theme contained 13 categories and 84 raw themes. There were four instances of categories being mirrored to motivate both physical activity and sedentariness. Overall, this higher-order-theme pertained to social interactions and relationships that directly facilitated/enabled, or restricted/prevented, physical activity.

#### Mirrored categories

Categories pertaining to the geography and the physical environment motivated both physical activity and sedentariness. Close proximity to facilities that were affordable and well-maintained were described as motivating physical activity, whereas lack of access – either by distance, price or other accessibility issues – and poor maintenance were described as discouraging activity and promoting sedentariness. Examples of these themes include:

I now work around 1.7 miles from where I live, so it’s just easier. And I mean it’s the bonus of not having to pay for parking as well. I just get out my door: no traffic, no ridiculous prices for parking… plus you get the health benefits _[M-37-Warehouse-Supervisor-PROXIMITY]_

I live in a country [rural] area where it’s just dangerous to go out [cycling or walking] in the dark. You’ve got cars, they’re not going to see you, you could have a severe accident._[M-34-Office-worker-RURAL LOCATION]_

We live out in the country, probably ten miles away from the nearest town, so, it’s just not the cheapest thing. We would do it for a little while but I know that very quickly we would be, you know, ‘Oh we can’t be bothered with the driving to and from’. There’s no question that because we live out in the country we lose out. If it was just around the corner I think it would be different. _[M-49-Manager-RURAL-LOCATION]_

Logistical/pragmatic support could either facilitate physical activity (e.g., “He looks after the kids while I go to the gym” _[F-35-Carer-HUSBAND]_), or its removal/absence could undermine it (e.g., “My partner could be more encouraging in terms of running the home and sorting the children out… I can’t go off and do gardening, if I wanted to I’d have to negotiate that” _[M-49-Manager-WIFE]_). Likewise special projects and initiatives could either motivate physical activity (when present) or sedentariness (when absent or ended). For example: “The NHS influenced me… with their campaign ‘Couch-to-5K’… I would have thought that influenced me a lot”_[F-35-Carer-GOVERNMENT]_ versus “SureStart did do a good job, we went on a woodland walk and went out into nature; the children liked it too. But they don’t do it anymore. It’s to do with funding… it’s all to do with funding”_[F-35-Carer-GOVERNMENT]_. Finally, participants made it extremely clear that ‘workload directly relates to physical activity’, such that increased work volumes or high work stress could easily undermine being active. In contrast, flexible working arrangements or self-employment were viewed as conducive to physical activity. As an example, the most active participant responded to the question ‘what would it take to make you sedentary, to stop you being active?’ as follows: “I suppose if my work hours increased then I couldn’t continue in the same way, or if I changed to a different type of work… I’d probably just be working hard and reading and watching television: more sedentary things”_[M-49-Manager-WORKLOAD]_. Another described how worked dominated his life:

It takes up the majority of my time. I commute for an hour and a half… it’s just *time*… Wake up: seven o’clock in the morning. Leave the house: ten to eight. Get to work around nine. Finish at 6, seven o’clock. By the time I get home it’s eight. Spend half an hour with my daughter. Cook tea or watch TV. Do the pots. Watch TV. Spend a bit of time with my missus. Then it’s off to bed. _[M-31-Sport-Centre-Manager-WORK-LIFE BALANCE]_

#### Non-mirrored categories

One category that motivated physical activity was not mirrored under promoting sedentariness: ‘happy coincidences’ reflected the possibility for unintended pathways into physical activity, such as a daughter wanting a dog then not taking it for walks. Four categories only motivated sedentariness: (i) Family and work always come first – which reflected the idea that any problem with family or work would immediately supplant physical activity (e.g., “I mean God forbid but if something happened and one of my children were ill, or seriously injured… then everything else goes out the window: my one single priority is my family”_[M-36-Warehouse-Supervisor-FAMILY]_, and “I would rather spend an hour-and-a-half with my daughter than doing exercise… even if it’s just sitting on the sofa cuddling her, I would rather do that because I haven’t seen her all day”_[M31-Sport-Centre-Manager-DAUGHTER]_) (ii) provision of facilities/opportunities – no gym/pool nearby, no crèche at gym, gym too expensive or memberships too long/inflexible; (iii) social events can undermine physical activity – such as one-off dinners or the visits of friends from out-of-town; and (iv) lack of targeted provisions (e.g., failure to target new mothers or parents of children with special needs).

### Relatedness and belonging

This higher order theme contained eight categories and 37 raw themes. There were three instances of categories being mirrored to motivate both physical activity and sedentariness. Overall, this higher-order-theme captured the co-dependency of physical activity and social relationships: either through one-to-one relationships or group membership.

#### Mirrored categories

Categories pertaining to ‘group membership’ motivated both physical activity and sedentariness. Being a member of a social group could motivate physical activity (e.g., “You’re motivated to go if there’s a group of you. It’s easier to say no if you’re on your own. It’s the social side as well, isn’t it? Not just in a gym pounding my legs” _[F-49-Sales-Assistant-EXERCISE CLASSMATES]_, and “I guess it’s just the commitment you make, and it’s not wanting to let other people down” _[M-31-Office-Worker-TEAMMATES]_). In contrast, this group membership could also motivate sedentariness (e.g., “There’s a group online saying ‘Oh come on, one more hour’ and that turns into two… then 6, can easily get to quite a few more hours. There’s a lot of people on it” _[M-34-Office-worker-ONLINE GAMING PEERS]_). Likewise, ‘opportunities to improve/expand social bonds’ could either motivate physical activity or sedentariness, depending whether those being bonded with were active or sedentary. Examples of these influences include: “It’s the social aspect as well, isn’t it? And I suppose keeping fit. Meeting other people… because I don’t know them… and then working as a team… team efforts”_[F-43-Teaching-Assistant-CLASSMATES]_ versus “But I met a lot of people on it [online gaming], and earlier on this year I actually met three of them. Thats also given me things to do because I can go across… go and see them.”_[M-34-Office-Worker-ONLINE-GAMING-PEERS]_.

Finally, the relationship with the instructor could either motivate physical activity or sedentariness, such that a poor relationship could lead to sedentariness, or a good relationship could motivate physical activity. The following quotes illustrate this influence: “It was mainly the way she spoke to the group, at the front, she was just quite comical and she’d have us in… she was good!”_[F-56-Office-Worker-INSTRUCTOR]_; *versus* “I didn’t look forwards to the abuse that personal trainers can dispense…. I’d probably start fighting… or start swearing…”_[F-47-Novellist-PERSONAL TRAINER]_.

#### Non-mirrored categories

Two categories that motivated physical activity were not mirrored under promoting sedentariness. These included: (i) “Do it for your family” – wherein family became a sufficient reason to pursue good health (e.g., “I’m a father and I want to be fit and healthy to provide for them”_[M-36-Warehouse-Supervisor-CHILDREN]_); and (ii) mutual pushing (e.g., “Well it’s like you support each other really, because I’m sort of keen, but he definitely pushes me, my colleague”_[F-45-Nurse-WORK COLLEAGUE]_).

### Commentary on socio-environmental influences

On reviewing Table 2 it is clear that the majority of the socio-environmental influences that were identified in the analysis could motivate either activity or sedentariness. Close family (wife, husband, partner, daughter, son, mother, father); friends, workmates, team and classmates, employers, strangers, personal trainers, general practitioners, media, governments and the physical environment could all motivate both forms of behavior. In contrast, social media, websites apps and podcasts, exercise referral workers and nurses, class instructors and siblings were only listed as motivating physical activity in this study. Likewise, online gaming peers and gym/companies were only listed as motivating sedentariness – perhaps because the services offered by gyms have certain assumptions and standards expected, and so only perceived faults/problems were noted by participants in this study. It is possible, indeed likely, that each type of socio-environmental influence can motivate both physical activity and sedentariness at different times, in different ways, as the data from this paper is unlikely to be exhaustive. Overall, the observed pattern suggests that – pending future investigations - there are unlikely to be any exclusive ‘angels’ or ‘devils’ amongst these socio-environmental influences, when it comes to motivating physical activity or sedentary behavior.

**Table 2:** Summary of the motivational influences towards either physical activity or sedentariness from each social agent

Social agent	Behaviors and attributes reported to motivate physical activity	Behaviors and attributes reported to motivate sedentary behavior
Husband/wife/partner	Allowing me time to be active, constantly prompting me to be active, role modeling PA behavior, doing activities together, encouraging me, reminds me of friends made through PA, looks after kids while I go.	Disinterested, does not approve, begrudges time spent away being active, is very sedentary him/herself, we are sedentary together (TV), works too much/late. Reminders can be demotivating. Reminders needed/appreciated. Could help by looking after kids while I am active.
Parents/father/mother	Setting a good example, prompting/asking, preferring to be outdoors, taking an interest and supporting, “showing my future”.	Prefer to take car which means I have to too.
Brother/Sister	Accompanying me, teaching me new skills, sibling rivalry	
Children/son/daughter	Helping him/her ends up making me active too, taking an interest in my PA, noticing my achievements, accompanying parent to gym, prompting/pushing parent to be active.	Looking after kids leaves me exhausted, being sick/requiring care prevents PA opportunities, I would rather spend time with them, insisting on sedentary activities (video games)
Family	Doing everything together (inc. PA), making PA our norm, letting me fit PA around them, being a reason to stay healthy.	Our routine is to watch TV in an evening, family comes before ‘personal’ activities, having children takes over your life, having no help to run household reduces PA opportunities, any illness/problem would have to come before PA.
Grand children	Requiring a lot of activity to look after	After caring for them I can’t face any PA.
Friend/Friends	Introducing me, recommending activities, being ‘sporty’, being another reason for me to attend, group membership/belonging, allowing me time alone too.	Being lethargic/sedentary meant I had to be if I wanted to be with them, being unable to walk far means parking closer, no friend to help/support me, we became friends through sedentary activities (gaming, drinking), socializing trumps PA (rarer/more important)
Online gaming peers		I made friends through online gaming, encouraging me to stay online (sed.) a bit longer.
Work-mates or study colleagues	Being active themselves, insisting on taking the stairs, recommending classes/activities, agreeing to do activities together, identifying a person as ‘active/sporty’, being in poor health as an example to avoid.	Not interested in PA.
Neighbors	Going for regular walks	
Strangers (adults, older, younger)	Showing me what is possible, building relationships during active commuting.	Being rude during PA – at gym or in sport, appearing to have negative attitude towards those who exercise.
Team-mates or opponents (sport)	Group membership/belongingness, asking me to return after I quit, using sport for social time, building friendships through sport, allowing quiet time when I need it. Boasting so I want to beat them.	Attaching social events (drinking) to training/games, quitting can scupper a team/group (e.g., if 4 needed); being too good, or appearing intolerant of beginners, making negative comments.
Gym Colleagues	Going as a pair creates commitment, leaving me alone when I need it.	
Gym Instructor	Helping find right equipment, teaching me the right technique, challenging me to races/goals	
Class Instructor	A great teacher, differentiates tasks, seeks gradual improvement not step changes	
Personal Trainer	Tailoring program to specific needs, personalizing program to disability, structuring program so progress is self-evident, challenging me to races/goals.	Shouting or using negative motivation
Exercise referral worker/health worker	Performing (and following up) lifestyle audit, referring me for extra treatment, helping me find and take opportunities to be active.	
General Practitioner	Diagnosing illness and detailing consequences, highlighting risk factors and detailing consequences, advising me to lose weight or reduce blood pressure, informing me of warning signs to avoid/manage, referring me for specialist help	No advice given implies I must be ok.
Practice Nurse	Referrals to exercise or weight management groups, being a bit stern so I fear negative judgment.	
Exercise class-mates	Creating a social bond and sense of belonging, needing my help to motivate them, pushing me – by sheer presence or deliberate/vocal	
Employer	Jobs that involve promoting PA and health, workplace schemes to be active, reducing workload or allowing flexible hours	High workload prevents PA, long hours prevent PA, workload direct inverse relationship to PA, work is more important than PA, Stress makes me not feel like PA, job involves being sedentary, design of workplace undermines PA, inconsistent work patterns so cannot join class/team; work plus family leaves no time for PA, no schemes or initiatives, long commute leaves no time.
Event organizers and community groups	Publicizing event details, allowing me to get involved by organizing sparked interest	
Gyms/companies		Too expensive, need flexible membership options, high expectations of beginners, over-crowded gym, no entry level activities, need a crèche, need classes targeting new mums.
Government	Incentives and schemes, information campaigns, parking charges	Restricting access to certain locations (reservoirs), poor public transport, removing funding from schemes that were working.
Media	Educating and informing, reinforcing advice from doctors (or good advice), bringing the issue straight into our home, showing extreme images and worst cases, promoting local events and initiatives, prompting me to see GP, providing a constant ‘nudge’	Inconsistent/contradictory messages, attention grabbing so I sit and read/watch, “so many opportunities”, shock tactics can desensitize me, promoting particular body image demotivates me, targeting certain groups over others. “TV is the main threat”
Cultural norms	“Slim women”, “muscular men”, “overweight=bad”	Its “just normal” to curl up in and evening and put the heating on, need to look smart for job (cannot get rained on or sweaty), simple gender roles dictate wife stays indoors.
Social media	Making us aware of upcoming events, sharing achievements	Telling us that everyone these days is inactive, making other people seem super-human
Websites, apps and podcasts	Information is helpful, recording calories, announces/publicizes my achievements	
Available activities	Suited to individual preferences (group/individual, competitive/classes)	Everything seems expensive, too far away = unable to travel, changing format of sessions put me off, pace was too high/low.
Physical Environment	Facilities are close by, converting disused land/facilities into PA opportunities.	No facilities in my area, transport costs too high, need to be within walking distance, not safe to cycle on these roads, quality of facilities is poor

### Commentary on mirrored themes

On reviewing Table 3, it is clear that many broad categories of behavior can motivate either physical activity or sedentariness (all the themes highlighted in *italic*). Thus we reach the finding that, for example, social relationships can form around pastimes that are either active or sedentary, or that simple approval and moral support in relation to certain tasks makes it easier for a close family member to pursue them (be they active or sedentary). Overall, it appears that the context, intention behind, and perceptions of specific behaviors are important. There are clearly instances of categories that exclusively motivated either physical activity or sedentariness, but focusing on these (e.g., reinforcing the former and extinguishing the latter) may be misguided. The majority of categories in these data were mirrored, suggested that the key is in using these existing mechanisms to motivate physical activity *instead of* sedentariness.

**Table 3:** Analysis of categories of behavior and their organization into five higher order themes

Motivating physical activity			Motivating sedentariness	
Category of behaviors/attributes	Social agents	Overall higher theme	Social agents	Category of behaviors/attributes
Healthy competition	Team-mate, opposition, siblings, instructors, son/daughter	Competence and progress	Strangers, Team-mates, class-mates	**Fear of negative social judgments
**Fear of negative social judgments	Nurse			
Noticing/recording progress	Son/daughter, Website/App, work colleagues			
Activities individualized to me	Website/app/podcast, personal trainer, gym instructor		Available activities, gyms/companies, government	**Nothing that suits me, that I want to do
Public accountability	Website/app, social media, work colleagues			
Realistic pace of progress	Health worker, class instructor			
Showing me how to do it properly	Gym instructor		Team-mates/class-mates	**Social comparisons (I compare badly)
**Social comparisons (I compare well)	Work colleagues, friends			
**The nature of the activity on offer has to suit me	Available activities			
“Happy coincidences” – facilitating PA by accident or shared interest	Social media, employer, family, work colleagues, event organizers	Pragmatics and logistics	Husband/wife/partner, Daughter/son, child, Grand-children, Family, Employer	Family and work always come above exercise/PA
**Beneficial geography or local area	Physical environment, government		Physical environment, government	**Geographical and local issues
			Wife/husband/partner, Team-mates	**Not supporting, or removing support
**Logistical/pragmatic support	Husband/wife/partner, family, gym instructor		Gyms/companies, physical environment, available activities	**Poor provision of facilities/opportunities
			Friends	Social events can undermine PA
**Special projects and initiatives	Government, physical environment, employer		Government, Employer	**Lack of special projects and initiatives
			Available activities, gyms/companies, government	**Lack of targeted/specialist provision
**Workload directly relates to PA	Employer, physical environment (commute)		Employer, Family, Physical Environment (commute)	**Workload directly relates to PA
**Consistency of messages between sources	Media, General Practitioner	Informational influences	Media, physical environment, available activities, family, friends, cultural norms	“Too many temptations” towards sedentariness
My job gives me awareness of key issues	Employers, work colleagues		Media	**Inconsistency of messages between sources
Personal assessments opened my eyes	Exercise referral worker		Media	**Demotivating, desensitizing, and making me feel hopeless
Raising awareness and ‘making me think’	Media, government		Employers (and clients), cultural norms, Media	**Norms promoting sedentariness
Referrals and recommendations	General practitioner, nurse, work colleagues			
**Role models and leading by example	Husband/wife/spouse, Parents, Brother/Sister, Strangers, neighbors, work colleagues, media		General practitioner	**Failing to raise the issue with me
**Shocking images/stories scare me	Media, government			
**Norms promoting physical activity	Friends, work/study colleagues, society/culture		Husband/wife/partner, Parent/father/mother, Family, Friend	**Their sedentariness limits what I can do
**Warning signs and alarm bells	GPs, media, family (e.g., older), friends getting sick			
Allowing “me time” – to do it, or during	Husband/wife, friends, Team-mates, gym colleagues	Emotional influences	Friends	**Looking after (or spending time) with sedentary friends
**Altruism – supporting each other	Exercise class-mates, daughter/son/child		Husband/wife/partner	**Lack of support/encouragement
**Moral support, encouragement, interest	Husband/wife/spouse, son/daughter/child, Parents, Website/App,		Husband/wife/partner, Parent/father/mother	**Constant reminders can demotivate
**Prompting/reminding - of PA or health	Daughter, Wife/partner, Father/mother, friends, team-mates		Husband/wife/partner, Parent/father/mother, family, Friend, online gaming peers	**We engage in sedentary behavior together
**We do activity together	Husband/wife/spouse, son/daughter/child, Parents, Brother/Sister, Friends, Classmates/Team-mates			
“Do it for your family” – being a reason to stay healthy	Children, family	Relatedness and belonging	Work colleagues, Family, Team-mates, Online gaming peers	**Group membership motivates sedentariness
“Mutual pushing” – doing it together and pushing each other	Wife/Husband/partner, Son/Daughter, Work colleague, team-mate		Personal trainer, gym instructor, class instructor	**Poor relationship with instructor
**Group membership in relation to PA fosters commitment	Team-mates, class mates, friends, strangers, husband/wife/partner			
**PA provides opportunities to improve/expand social bonds	Parent/father/mother, husband/wife/partner, team-mates, class-mates		Online gaming peers, Husband/wife/partner, Family, Friends	**Social network supports sedentariness
**Good relationship with instructor	Personal trainer, gym instructor, class instructor			

Mirrored themes are highlighted with italic font and a (**) symbol. Social agents are paired with the categories of behavior for illustration. Each category may contain between 2 and 20 raw themes

## Discussion

This study employed qualitative methods with working age adults to establish: (i) the socio-environmental influences motivating either physical activity or sedentary behavior; and (ii) specific behaviors and attributes that were perceived to influence motivation. Additionally we set out to develop a framework to analyze and interpret those motivationally relevant behaviors, which may inform future research and practice. Findings focused on five broad themes of motivational influence: (a) competence and progress; (b) informational influences; (c) emotional influences; (d) pragmatics and logistics; and (e) relatedness and belonging. Each of these themes related to the motivation of both physical activity and sedentary behaviors, with many categories within the analysis reported to motivate both types of behavior. Likewise, almost all the socio-environmental influences identified in the study were reported to motivate both physical activity and sedentariness in different instances. A comprehensive list of socio-environmental factors was generated, and these sources-of-influence were associated with particular motivationally relevant behaviors.

### Comparison to existing theory and research

While attempts were made to minimize the influence of existing theories in determining what to study, how, and how to interpret results (see Introduction), it is still important to reconcile the current findings with existing knowledge. The categories and themes identified in this study are consistent with existing theoretical and empirical knowledge, as well as offering new insights. For example, self-determination theory [63] posits that ‘optimally’ motivating social environments support the psychological needs to experience competence, autonomy and relatedness. The five high-order themes identified in this study can be readily aligned to this theory. Supporting competence is addressed in the theme of ‘competence and progress’. Supporting autonomy is addressed by the themes of informational influences, emotional influences, and pragmatics and logistics – particularly when exploring the categories and raw themes. Likewise, supporting feelings of relatedness can be achieved by examining the themes from ‘relatedness and belonging’. Separately, social support theory describes the degree to which people have assistance available from other others, and that they are part of a supportive social network [64]. Key dimensions of social support are: (1) emotional support (e.g. comfort, validation, ‘there for you’); (2) informational support (e.g. advice and guidance); (3) tangible support (also known as material or instrumental support - e.g. concrete assistance such as purchasing equipment and providing transport); and (4) esteem support (bolstering self-confidence and providing reassurance [65, 66]. The findings of this study can be mapped onto social support theory: with competence and progress reflecting ‘esteem support’; informational influences reflecting ‘informational support’, emotional influences reflecting emotional support, and pragmatics and logistics reflecting ‘tangible support’. Only the theme of ‘relatedness and belonging’ is less immediately reconciled to social support theory. As such, the findings of the current study are credible in relation to existing literature, but also expand current knowledge by identifying specific socio-environmental factors and the specific behaviors that influence motivation towards physical activity. Hence, for example, where perceptions of ‘information social support’ are shown to predict behavior change or intervention adherence, the current findings offer specific behavioral recommendations to create those perceptions. Such informational support may include: personal assessments and individual health advice from doctors and nurses; awareness-raising, education, and (in some instances) shocking stories/images in the media; referrals between health practitioners, and role-modeling of ideal behaviors (friends, family, work colleagues). On the issue of warnings (from health professionals) and shocking images in the media, a review of intervention studies demonstrated equivocal findings [67]. Specifically, significant interactions have been shown between threatening communications and efficacy, such that threats were only effective when the recipient felt able to change their behavior or lifestyle. This finding is consistent with the message of this study that socio-environmental motivators of physical activity (and sedentary behavior) are complex and interactive. Thus if it were decided to use a health professional or the media to introduce a threatening message, the recipient should also be supported and enabled to pursue behavior change.

In relation to the trans-theoretical model of behavior change [68], and its application to physical activity and/or sedentary behavior, the findings of this paper also offer practical insights. In this approach to behavior change, participants are classified into a ‘stage-of-change’ in relation to reaching and maintaining the desired behavior (i.e., pre-contemplation, contemplation, preparation, action and maintenance). Different processes-of-change are associated with each stage, and the current finding offer insights as to which social agents can perform which specific behaviors for each process. For example: (i) *consciousness-raising* was reflected in the themes of ‘informational influences’ such as media campaigns, assessment by health practitioners, and role-modeling by family and friends; (ii) *dramatic relief* would also be closely aligned to media and primary health practitioners (for the initial ‘bad news’) followed by friends, family and health/fitness providers to provide the relief; (iii) *self-re-evaluation* could be prompted by general practitioners and nurses as well as wearable devices and web-apps, and also friends or family ‘leading by example’; (iv) *environmental re-evaluation* - realizing one’s effects and dependence on others – would require reasonably close connections with family or friends to form the frame of reference, and is well represented under the themes of ‘relatedness and belonging’ (e.g., support for being active or sedentary) as well as ‘informational influences’ (norms and expectations around activity or sedentariness); (v) *social liberation* - realizing that society is more supportive of the healthy behavior – appears to be well supported by both team- and class-mates, as well as gym-instructors, exercise leaders and family members (note here that primary health professionals and media appear less well-suited to these categories); (vi) *self-liberation* appears to be closely aligned to themes under ‘pragmatics and logistics’ in the current data, and may be provided chiefly by family, friends, employers, local government and the organizers of local activities or groups; (vii) *helping relationships* are well addressed under ‘relationships and belonging’, once again with family, team- and class-mates, and instructors/trainers being key sources of influence. (viii) *Counter-conditioning* (substituting unhealthy ways of acting and thinking for healthy ways) and (ix) *reinforcement management* (increasing the rewards that come from positive behavior and reducing those that come from negative behavior) are two themes that were not well-discussed by participants – perhaps because these may apply more at the personal level (especially of the aims of these stages is to increase intrinsic motivation rather than extrinsic rewards - [69]). (x) *Stimulus control* - using reminders and cues that encourage physical activity – appeared to be managed either by close family and friends (including team- and class-mates), or personal devices and web-apps. Perhaps outside the stage-of-change model, the physical environment – built and natural – as well as government policies, are arguably some of the most pervasive and influential stimulus control influences. The counterpoint to all of the above is that similar sources and forms of motivational influence were described in regard to sedentary behavior. This indicates the possibility of a less readily detected (and thus resisted), pathway into sedentary behavior; where none of the ‘stages-of-change’ – towards the undesirable outcome of sedentariness - are deliberate or conscious (see also [70]). If such influences were to prove less readily recognized and resisted, it would require a different methodology, as interviews only permit the reporting of phenomena that can be noticed and articulated by participants. Nonetheless, in the mean time our findings offer traction to those seeking to implement a specific process of change: offering both potential behaviors as well as appropriate agents to enact those behaviors. Finally, these findings may be helpful in reference to the Theory of Reasoned Action [71, 72], specifically in relation to the ‘elicitation’ procedure prescribed for successfully applying the theory. Kok and Ruiter ([73]; p.62) noted that “It is… surprising how many authors apply [this theory] without the careful elicitation of beliefs through adequate qualitative and quantitative research, a procedure which Fishbein and Ajzen proscribe as an essential prerequisite for application”. The findings of the present study may inform the elicitation of social norms/values as well as their influences/sources – i.e., what to look for, and from whom. Where such beliefs and intentions are modifiable, the present findings may offer added insight into how to approach this. Hence, despite adopting a theoretically agnostic inductive approach [35, 36], the findings of this study are reconcilable with several existing and relevant theories: self-determination theory, social support theory, the trans-theoretical model and the theory of reasoned action. The present study worked on the assumption that using a particular theory as a guide, prescribing what to look for, how to look and how to interpret the findings may generate data that are only reconcilable with, and aligned to, the tenets of one theory. One benefit of this approach is that researchers working to apply or evaluate any one of these theories could draw on the current findings to inform their interventions.

### Comparison to other populations

Work aged adults are a relatively under-studied population precisely because their social influences are unpredictable. Workplaces, family arrangements, and lifestyle choices can vary substantially between working adults. In contrast, children almost universally attend school and older adults are more frequently engaged in the healthcare system. Hence, for example, Brustad’s review [74] on the social influence on children’s motivation towards physical activity identified only parents, school and the physical environment (access/opportunity) as core sources of influence. In contrast, work aged adults experienced a significantly more diverse range of socio-environmental influences. As such studying this population in the first place is a strength of the current study. Despite the more diverse sources of influence, when compared against the broad categories of influence in children (i) promoting competence; (ii) supporting autonomy through social support (pragmatic, emotional and informational); and (iii) the importance of relationships and group membership, are all clearly echoed in recent reviews of motivational climate/atmosphere for children [29, 31, 39].

On the basis of these findings and other recent papers, methodologies based on realist assumptions and acknowledging complexity, may hold promise for the study of motivational climates. Much of our current understanding of motivational climates is based on the *a priori* adoption of theory-derived questionnaires, which assess relatively broad perceptions of the social environment [30]. These studies are valuable in demonstrating that when people perceive their social environment supports particular concepts, such as autonomy, their motivation benefits. The question of precisely how these perceptions are generated, however, cannot be readily answered by assessing such generalized subjective perceptions, and this was a central consideration in adopting critical realist assumptions for this study [33, 62]. By generating a list of the raw ingredients in adults’ social-motivational eco-system, this study informs future research that seeks to motivate physical activity and reduce sedentariness. Additionally, while valuable findings this area have come from focusing on the social influences of specific social agents (e.g., parents [75], grandparents [76], physical environment [77, 78]), there may be additional insight available from examining concurrent motivational influences. By studying multiple sources-of-influence concurrently, it becomes possible to identify interactions, co-dependencies and recurring themes. For example, the concurrent role of children and partners in the family, referrals between health professionals, and the importance of consistent messaging between the media, government and health professionals were all emphasized in these data.

#### Limitations

It is important to remain cognizant that this study merely sought to generate a list of the key socio-environmental influences on motivation towards physical activity, and then explore how this occurs. The broad scope of the study meant that it was not possible to establish the specific ways that large groups/categories of behaviors combine to influence motivation, and this should be addressed in future studies adopting a tighter focus and perhaps a different methodology. Likewise, the study was limited as the sample contained many more white/British participants than other ethnicities and nationalities, specifically focusing on the North East of England. As such these findings may not be generalizable across all contexts. However, differences between genders, ethnicities and nationalities were not a focus of this study, and future large-scale or quantitative studies may be able to explore any such differences. Other limitations of the study include reliance of the qualitative methodology on participants’ recall and ability to articulate their experiences effectively. For example, the results of this type of study reflect what people themselves see as influencing their motivation, which may overlook any influences that participants were unable to perceive or articulate (on reflection, we would speculate that this phenomenon is more applicable to the factors promoting sedentariness than activity, as people were often less fluent and eloquent in response to those questions). Whilst the quality and depth of the responses provided would suggest these were not serious problems, they must be considered in evaluating the findings of the study. Additionally, on reflection, there may still be examples of motivationally relevant social influence that were not explicitly specified by participants in this study. The existence of non-mirrored themes and the sampling of data from one geographical region both suggest that the resulting model still has space for additional clarification (i.e., a substantive model as opposed to a formal theory [79, 80]). Future replications, for example in different contexts or cultures, may identify additional raw themes and lead to a model that may be generalized across contexts and cultures. The higher-order themes may be less likely to require updating than raw themes, and it seems reasonable to expect counter-instances of some non-mirrored themes. Thus when interpreting these findings, absence of evidence should not be interpreted as evidence of absence.

#### Implications for applied practice

The finding of this paper may inform the intake and needs analysis processes of health practitioners, on the occasions they assess the physical activity profiles of work aged adults. Indeed, one participant reported that the ‘lifestyle audit’ performed by the practice nurse formed the touchstone around which her lifestyle changes could be planned and monitored _[F-57-Retired-EXERCISE-REFERRAL-WORKER]_. We know from previous questionnaire-based research that, for example, perceiving one’s psychological needs are being supported consistently predicts subsequent motivation and behavior [81]. The current study enables such finding to be translated into practice, detailing both the places to look for such support and interventions that might be recommended. In light of the findings of this study, practitioners may be able to direct patients to specific sources of competence evaluation, information and education, emotional support, pragmatic/material support, or social relatedness. For example, the general practitioner is a good source of information regarding diagnoses, management and prognoses, but may not be a typical source of friendship or logistical influences. Immediate family (spouse, children, parents) and friends (including team-mates or class-mates) appear to be more typical sources of emotional and logistical influences. It may even be possible to create a new role, not currently captured in the social network, of physical activity advocate. These specialists could receive referrals from primary health-care and perform the above audit of social influences, offer guidance, counseling and planning support, and connect patients directly to the most appropriate local opportunities/organizations.

## Conclusions

  This paper adopted an exploratory approach to identifying the social influences on motivation towards either physical activity or sedentariness in work aged adults: a relatively under-studied yet important population. A wide range of social agents were reported to influence this motivation, through an even wider range of motivationally relevant behaviors. Consistent with the core assumptions of the paper, the findings suggest a complex and interactive ‘motivational atmosphere’, wherein a the impact of a single behavior is not simple and direct, but depends on its source and context. The findings of this study are both consistent with prior research but also offer opportunities to expand current knowledge, and improve the effectiveness of interventions seeking to promote physical activity in work-aged adults. One final conclusion from this study emerges as a result of examining the specific behaviors, from specific socio-environmental agents, in determining motivation towards important lifestyle choices. Upon adopting this methodology, it become clear that we are dealing with a complex system, as opposed to a complicated one [31, 82, 83]. As such, our methods – both in research and in practice - may need to adapt to accommodate this complexity. With the emerging availability of wearable technology, constant connection to the Internet, and new analytic tools such as agent-based modeling and neural networks, the required adaptations are increasingly within our reach.

## References

[1] World Health Organization. World Health Statistics 2011. WHO Libr Cat Data. 2011. doi: 978 92 4 156419 9.

[2] World Health Organization (2010). Global recommendations on physical activity for health.

[3] Dumith SC, Hallal PC, Reis RS, Kohl HW (2011). Worldwide prevalence of physical inactivity and its association with human development index in 76 countries. Prev Med (Baltim).

[4] Kohl HW, Craig CL, Lambert EV (2012). The pandemic of physical inactivity: global action for public health. Lancet.

[5] Zhang J, Chaaban J (2013). The economic cost of physical inactivity in China. Prev Med (Baltim).

[6] Carlson SA, Fulton JE, Pratt M, Yang Z, Adams EK (2014). Inadequate physical activity and health care expenditures in the United States. Prog Cardiovasc Dis.

[7] Yee SL, Williams-Piehota P, Sorensen A, Roussel A, Hersey J, Hamre R (2006). The nutrition and physical activity program to prevent obesity and other chronic diseases: monitoring progress in funded states. Prev Chronic Dis.

[8] National Institute for Health and Clinical Excellence. Physical Activity and the Environment. NICE Public Heal Guid; 2008. 56

[9] British Heart Foundation National Centre (BHFNC) for Physical Activity and Health, BHF National Centre (2013). Economic costs of physical inactivity.

[10] McCormick B, Stone I (2007). Economic costs of obesity and the case for government intervention. Obes Rev.

[11] Troiano RP, Berrigan D, Dodd KW, Mâsse LC, Tilert T, Mcdowell M (2008). Physical activity in the United States measured by accelerometer. Med Sci Sports Exerc.

[12] Sisson SB, Katzmarzyk PT (2008). International prevalence of physical activity in youth and adults. Obes Rev.

[13] Hallal PC, Andersen LB, Bull FC (2012). Global physical activity levels: Surveillance progress, pitfalls, and prospects. Lancet.

[14] Lee IM, Shiroma EJ, Lobelo F (2012). Effect of physical inactivity on major non-communicable diseases worldwide: an analysis of burden of disease and life expectancy. Lancet.

[15] Koeneman MA, Verheijden MW, Chinapaw MJM, Hopman-Rock M (2011). Determinants of physical activity and exercise in healthy older adults: a systematic review. Int J Behav Nutr Phys Act.

[16] Trudeau F, Shephard RJ (2008). Physical education, school physical activity, school sports and academic performance. Int J Behav Nutr Phys Act.

[17] Kohl HW, Cook HD (2013). Approaches to Physical Education in Schools.

[18] Horne M, Tierney S (2012). What are the barriers and facilitators to exercise and physical activity uptake and adherence among South Asian older adults: a systematic review of qualitative studies. Prev Med (Baltim).

[19] Van Cauwenberg J, De Bourdeaudhuij I, De Meester F, Van Dyck D, Salmon J, Clarys P, et al. Relationship between the physical environment and physical activity in older adults: a systematic review. Health Place. 2011;17:458–69. 10.1016/j.healthplace.2010.11.010 21257333

[20] Chogahara M, O’Brien Cousins S, Wankel M (1998). Social influences on physical activity in older adults: a review. J Ageing Phys Act.

[21] Sun F, Norman IJ, While AE (2013). Physical activity in older people: a systematic review. BMC Public Health.

[22] Keegan RJ, Biddle JH, Lavallee DE (2010). It’s not how old you are, it’s where you’re at in life : application of a life-span interventions framework to physical activity in examining community and environmental interventions. Sport Exerc Psychlogy Rev.

[23] Goldberg JH, King AC (2007). Physical activity and weight management across the lifespan. Annu Rev Public Health.

[24] Kirk SFL, Penney TL, McHugh TLF (2010). Characterizing the obesogenic environment: the state of the evidence with directions for future research. Obes Rev.

[25] Epstein LH, Roemmich JN (2001). Reducing sedentary behavior: role in modifying physical activity. Exerc Sport Sci Rev.

[26] Vallerand R, Thill E, Vallerand RJ, Thill EE (1993). Introduction au concept de motivation. Introd. à la Psychol. la Motiv. Editions études vivantes – Vigot, Quebec.

[27] Roberts GC, Singer R, Murphy M, Tennant K (1993). Motivation in sport: Understanding and enhancing the motivation and achievement of children. Handb. Res. Sport Psychol. New York MacMillan.

[28] Ames C (1992). Achievement goals and the classroom motivational climate. Student Perceptions Classr.

[29] Keegan R, Spray C, Harwood C, Lavallee D (2010). The motivational atmosphere in youth sport: coach, parent, and peer influences on motivation in specializing sport participants.

[30] Keegan R, Spray C, Harwood C, Lavallee D, Geranto BD (2011). From “motivational climate” to “motivational atmosphere”: a review of research examining the social and environmental influences on athlete motivation in sport. Sport Psychol.

[31] Keegan RJ, Spray CM, Harwood CG, Lavallee DE (2014). A qualitative synthesis of research into social motivational influences across the athletic career span. Qual Res Sport Exerc Heal.

[32] Archer M, Bhaskar R, Collier A, Lawson T, Norrie A (1998). Critical realism. Science (80-).

[33] Tilley N, Pawson R (2000). Realistic evaluation: an overview. Br J Sociol.

[34] Strauss A, Corbin J (2008). Basics of qualitative research: techniques and procedures for developing grounded theory. Basics Qual Res Tech Proced Dev Grounded Theory.

[35] Henwood K, Pidgeon N (2003). Grounded theory in psychological research. Qual Res Psychol Expand Perspect Methodol Des.

[36] Sandelowski M (1993). Theory unmasked: the uses and guises of theory in qualitative research. Res Nurs Health.

[37] Dey I (1999). Grounding grounded theory: guidelines for qualitative inquiry.

[38] Keegan RJ, Harwood CG, Spray CM, Lavallee D (2014). A qualitative investigation of the motivational climate in elite sport. Psychol Sport Exerc.

[39] Keegan RJ, Harwood CG, Spray CM, Lavallee DE (2009). A qualitative investigation exploring the motivational climate in early career sports participants: coach, parent and peer influences on sport motivation. Psychol Sport Exerc.

[40] Hagger MS, Chatzisarantis NLD (2015) The Trans-Contextual Model of Autonomous Motivation in Education: Conceptual and Empirical Issues and Meta-Analysis. Rev Educ Res 0034654315585005– 10.3102/0034654315585005 PMC4873731 27274585

[41] Salmon J, Owen N, Crawford D, Bauman A, Sallis JF (2003). Physical activity and sedentary behavior: a population-based study of barriers, enjoyment, and preference. Health Psychol.

[42] Hagger MS, Keatley DA, Chan DCK, Chatzisarantis NLD, Dimmock JA, Jackson B, et al. The goose is (half) cooked: a consideration of the mechanisms and interpersonal context is needed to elucidate the effects of personal financial incentives on health behaviour. Int J Behav Med. 2014;21:197–201. 10.1007/s12529-013-9317-y 23613325

[43] Hagger MS, Chatzisarantis NLD, Harris J (2006). The process by which relative autonomous motivation affects intentional behavior: comparing effects across dieting and exercise behaviors. Motiv Emot.

[44] Hagger MS, Chatzisarantis NLD (2014). An integrated behavior change model for physical activity. Exerc Sport Sci Rev.

[45] Lincoln G (1985). How can the naturalist meet these trustworthiness criteria. Nat. Inq.

[46] Morgan DL (1998). Practical strategies for combining qualitative and quantitative methods: applications to health research. Qual Health Res.

[47] Patton M (1990). Qualitative evaluation and research methods. Qual Eval Res Methods.

[48] Patton MQ (2002). Qualitative research and evaluation methods. Qual Inq.

[49] Pope C (2001). Qualitative research methods: a health focus: PL Rice, D Ezzy. Oxford: Oxford University Press, 1999, pp. 291. ISBN: 0 195 50610 3. Int J Epidemiol.

[50] Liamputtong P, Ezzy D (1999). Qualitative research methods: a health focus.

[51] Dew K (2007). A health researcher’s guide to qualitative methodologies. Aust N Z J Public Health.

[52] Sandelowski M (2004). Using qualitative research. Qual Health Res.

[53] Goulding C (2005). Grounded theory, ethnography and phenomenology: a comparative analysis of three qualitative strategies for marketing research. Eur J Mark.

[54] Cook KE (2005). Using critical ethnography to explore issues in health promotion. Qual Health Res.

[55] Roberts GC, Roberts GC, Treasure DC (2012). Motivation in sport and exercise from an achievement goal theory perspective: after 30 years, where are we?. Adv. Motiv. Sport Exerc.

[56] Strauss A, Corbin J. Grounded Theory Designs. Basics Qual Res Tech Proced Dev. 1998. grounded theory

[57] Tracy SJ (2010). Qualitative quality: eight “Big-Tent” criteria for excellent qualitative research. Qual Inq.

[58] Côté J, Salmela JH, Baria A, Russell SJ (1993). Organizing and interpreting unstructured qualitative data. Sport Psychol.

[59] Qualitative Data Analysis Software | Mixed Methods Research | NVivo. http://www.qsrinternational.com/product. Accessed 5 Jul 2015.

[60] LeCompte MD, Goetz JP (1982). Problems of reliability and validity in ethnographic research. Rev Educ Res.

[61] Biddle SJH, Markland D, Gilbourne D, Nikos LD, Sparkes AC, Chatzisarantis NLD (2011). Research methods in sport and exercise psychology: quantitative and qualitative issues. J Sports Sci.

[62] Pawson R, Tilley N (1997). An introduction to scientific realist evaluation. Eval. 21st century A Handb.

[63] Ryan RM, Deci EL (2000). Self-determination theory and the facilitation of intrinsic motivation, social development, and well-being. Am Psychol.

[64] Seeman TE, Berkman LF (1988). Structural characteristics of social networks and their relationship with social support in the elderly: who provides support. Soc Sci Med.

[65] Cohen S, Mermelstein R, Kamarck T, Hoberman HM (1985). Measuring the functional components of social support. Soc. Support Theory, Res. Appl.

[66] Rees T, Hardy L (2004). Matching social support with stressors: effects on factors underlying performance in tennis. Psychol Sport Exerc.

[67] Peters G-JY, Ruiter R a C, Kok G (2012). Threatening communication: a critical re-analysis and a revised meta-analytic test of fear appeal theory. Health Psychol Rev.

[68] Prochaska JO, Velicer WF (1997). The transtheoretical model of health behavior change. Am J Heal Promot.

[69] Deci EL, Ryan RM (2008). Self-determination theory: a macrotheory of human motivation, development, and health. Can Psychol Can.

[70] Maitland C, Stratton G, Foster S, Braham R, Rosenberg M (2014). The Dynamic Family Home: a qualitative exploration of physical environmental influences on children’s sedentary behaviour and physical activity within the home space. Int J Behav Nutr Phys Act.

[71] Fishbein M, Ajzen I (2010). Predicting and changing behaviour: the reasoned action approach. New York Psychol Press.

[72] Fishbein M, Ajzen I (1975). Belief, Attitude, Intention and Behaviour: An Introduction to Theory and Research. Read MA AddisonWesley.

[73] Kok G, Ruiter RAC (2014). Who has the authority to change a theory? Everyone! A commentary on Head and Noar. Health Psychol Rev.

[74] Brustad RJ (2012). Children’s motivation for involvement in physical activity. Oxford Handb Exerc Psychol.

[75] Johnson L, Chen T-A, Hughes SO, O’Connor TM (2015). The association of parent’s outcome expectations for child TV viewing with parenting practices and child TV viewing: an examination using path analysis. Int J Behav Nutr Phys Act.

[76] Li B, Adab P, Cheng KK (2015). The role of grandparents in childhood obesity in China - evidence from a mixed methods study. Int J Behav Nutr Phys Act.

[77] Henderson KE, Grode GM, O’Connell ML, Schwartz MB (2015). Environmental factors associated with physical activity in childcare centers. Int J Behav Nutr Phys Act.

[78] Smith AL, Troped PJ, McDonough MH, DeFreese JD (2015). Youth perceptions of how neighborhood physical environment and peers affect physical activity: a focus group study. Int J Behav Nutr Phys Act.

[79] Glaser BG, Strauss AL (1967). The discovery of grounded theory. Int J Qual Methods.

[80] Glaser BG (2008). Conceptualization: on theory and theorizing using grounded theory. Int J Qual Methods.

[81] Chatzisarantis NLD, Hagger MS, Smith B (2007). Influences of perceived autonomy support on physical activity within the theory of planned behavior. Eur J Soc Psychol.

[82] Miller JH, Page SE (2009). Complex adaptive systems: an introduction to computational models of social life: an introduction to computational models of social life.

[83] Eagle N, Pentland A (2005). Reality mining: sensing complex social systems. Pers Ubiquitous Comput.

